# Supplementary material for: Effects of Extraction and Evaporation Methods on Physico-Chemical, Functional, and Nutritional Properties of Syrups from Barhi Dates (Phoenix dactylifera L.)
Source: Foods. 2023 Mar 16;12(6):1268. doi: 10.3390/foods12061268 (PMC10048268; doi:10.3390/foods12061268)
Supplement: Supplementary file 1 [file foods-12-01268-s001.zip › foods-2251989-supplementary.pdf]

Supplementary Material

Effects of Extraction and Evaporation Methods on Physico-chemical, Functional, and Nutritional Properties of Syrups from Barhi Dates (*Phoenix dactylifera L.*)

Table S1. Characteristics of subjects participating in *in vivo* study

**Characteristics**	**Value**†
Gender‡	
Male	4 (36.36)
Female	7 (63.64)
Age (yr)	28.73 ± 3.50 (23, 34)
Weight (kg)	54.93 ± 6.86 (45.70, 67.00)
Height (m)	1.62 ± 0.06 (1.55, 1.72)
Body mass index (kg/m^2)	20.87 ± 1.32 (18.54, 22.65)
Fasting blood glucose (mg/dL)	91.55 ± 3.72 (84, 97)

†Means ± standard deviations of 11 subjects (n = 11) with minimum and maximum in parentheses;

‡Frequency with percentage in parentheses
